# Abnormal Ferroptosis in Myelodysplastic Syndrome

**DOI:** 10.3389/fonc.2020.01656

**Published:** 2020-09-02

**Authors:** Qi Lv, Haiyue Niu, Lanzhu Yue, Jiaxi Liu, Liyan Yang, Chunyan Liu, Huijuan Jiang, Shuwen Dong, Zonghong Shao, Limin Xing, Huaquan Wang

**Affiliations:** Department of Hematology, General Hospital, Tianjin Medical University, Tianjin, China

**Keywords:** myelodysplastic syndrome, ferroptosis, decitabine, apoptosis, pyroptosis

## Abstract

**Background:**

Ferroptosis is a form of iron-dependent non-apoptotic cell death, with characteristics of loss of the activity of the lipid repair enzyme, glutathione (GSH) peroxidase 4 (GPX4), and accumulation of lethal reactive lipid oxygen species. The mechanism of ferroptosis in myelodysplastic syndrome (MDS) is unclear.

**Methods:**

Cell viability assay, reactive oxygen species (ROS) assay, GSH assay, and GPX activity assay were performed to study the regulation of ferroptosis in MDS cells obtained from MDS patients, the iron overload model mice, and cell lines.

**Results:**

The growth-inhibitory effect of decitabine could be partially reversed by ferrostatin-1 and iron-chelating agent [desferrioxamine (DFO)] in MDS cell lines. Erastin could increase the cytotoxicity of decitabine on MDS cells. The level of GSH and the activity of GPX4 decreased, whereas the ROS level increased in MDS cells upon treatment with decitabine, which could be reversed by ferrostatin-1. The concentration of hemoglobin in peripheral blood of iron overload mice was negatively correlated with intracellular Fe^2+^ level and ferritin concentration. Iron overload (IO) led to decreased viability of bone marrow mononuclear cells (BMMNCs), which was negatively correlated with intracellular Fe^2+^ level. Ferrostatin-1 partially reversed the decline of cell viability in IO groups. The level of GSH and the activity of GPX4 decreased, whereas the ROS level increased in BMMNCs of IO mice. DFO could increase the level of GSH. Ferrostatin-1 and DFO could increase the GPX4 activity of BMMNCs in IO mice. Ferrostatin-1 could significantly reverse the growth-inhibitory effect of decitabine in MDS patients. Decitabine could significantly increase the ROS level in MDS groups, which could be inhibited by ferrostatin-1 or promoted by erastin. Ferrostatin-1 could significantly reverse the inhibitory effect of decitabine on GSH levels in MDS patients. Erastin combined with decitabine could further reduce the GSH level. Erastin could further decrease the activity of GPX4 compared with the decitabine group.

**Conclusion:**

Ferroptosis may account for the main mechanisms of how decitabine induced death of MDS cells. Decitabine-induced ROS raise leads to ferroptosis in MDS cells by decreasing GSH level and GPX4 activity.

## Introduction

Cell death is a critical event in normal development, homeostasis, and prevention of cancer. It is tightly connected with other biological processes reflecting vital physiological and pathological changes in cells. The Nomenclature Committee on Cell Death has recognized and designated the distinction between accidental cell death and regulated cell death. The accidental cell death is triggered by severe physical, chemical, and mechanical insults and cannot be reversed by molecular perturbations, whereas regulated cell death is a programmed process that can be modulated pharmacologically and genetically and is therefore controllable (1). For a long time, it has been widely accepted that apoptosis is the only form of programmed cell death, whereas necrosis serves as a synonym for accidental cell death. However, in recent years, this traditional notion has been challenged by the discovery of several unrecognized, regulated cell death forms, including necroptosis, ferroptosis, autophagy, and pyroptosis. Critical signaling pathways have been identified for some of these regulated non-apoptotic forms of cell death, such as necroptosis, which is found to be induced by tumor necrosis factor, whereas many other cell death forms are still under extensive investigation (2).

Ferroptosis is identified as a regulated process of cell death featured by iron-dependent lipid peroxidation, which is completely different from all known forms of cell death, as it has distinct morphologic and biologic features and is modulated by a distinct set of genes. It is generally believed that excessive iron mainly causes iron death through active oxygen produced by Fenton reaction (3). Lipid peroxide is the executor in the process of iron death. Phosphatidylethanolamine is the preferred substrate for lipid oxidation. Glutathione peroxidase (GPX) 4 is a key enzyme that regulates iron death. It can catalyze the reduction of lipid peroxides to inhibit the occurrence of iron death. Inhibitions of GPX4 and system Xc^–^ are two ferroptosis-triggering mechanisms. Inhibition of system Xc^–^, the cystine-glutamate antiporter, suppresses cellular uptake of cysteine and impairs the production of glutathione (GSH) (4, 5). GSH is a cofactor for GPX4, which is an essential antioxidant enzyme exerting detoxication function by eliminating diverse lipid peroxides in cells. GPX4 uses GSH as a reducing enzyme via its peroxidase reaction cycle. Therefore, inhibition of system Xc^–^ indirectly suppresses GPX4, which leads to the accumulation of lethal lipid reactive oxygen species (ROS) and initiation of ferroptosis. Erastin, sulfasalazine, and sorafenib are small molecules that induce ferroptosis through this mechanism (4, 6). Direct inhibition of GPX4 also triggers ferroptosis in sensitive cells, which cannot eliminate the accumulation of lipid peroxides. Ferroptosis could be inhibited by iron chelation or ferrostatin-1 (Fer-1), a small molecular inhibitor of ferroptosis that prevents the erastin-induced accumulation of lipid ROS. Studies are investigating the lipid peroxides using a generic lipophilic ROS sensor (e.g., BODIPY-C11) (4, 7), but the identity of the lipid species that are oxidized during ferroptosis and drive cell death is still unknown. Identifying these lipid oxidation events in the process of ferroptosis may provide more insights into the mechanism of GPX4-regulated ferroptosis (4, 8). Studies have shown that inhibition of ferroptosis displays protective effects against neurotoxicity (9–11), whereas, on the other hand, data are indicating that certain tumor cells depend on GPX4 and system Xc^–^, such as hepatocellular carcinoma (12, 13), pancreatic tumor (14, 15), breast cancer (16, 17), renal tumor (7, 18), and B cell lymphoma cells (7); therefore, inducing ferroptosis may represent novel strategies for anticancer treatment (4).

Myelodysplastic syndrome (MDS) comprises a heterogeneous group of myeloid malignancies characterized by dysplasia in one or more cell lineages, ineffective hematopoiesis, and high-risk predisposition to leukemic transformation (19, 20). Resulting from an intermittent blood transfusion and ineffective hematopoiesis (21), 50–80% MDS patients eventually develop iron overload. Because ferroptosis is a distinct form of regulated cell death dependent on iron stores, it may participate in the occurrence and development of MDS, which may also be a new target for treatment.

Decitabine is one of the standard drugs approved for the treatment of relatively high-risk MDS. It is generally believed that the mechanism of decitabine in the treatment of MDS is demethylation. That is, it inhibits DNA methyltransferase (DNMT) and reverses methylation of the promoter region of tumor suppressor genes, thus suppressing the proliferation of cancer cells (22). However, a study by Flotho et al. (23) showed that the effect of DNMT inhibitors in leukemia cells was not related to DNA promoter demethylation, suggesting that demethylation drugs may also function through other pathways. Fandy et al. (24) showed that decitabine could induce ROS accumulation in leukemia cells, and active oxygen scavenger could decrease cell death induced by decitabine. Therefore, the effect of decitabine on MDS cells may also be related to ROS accumulation, given the fact that iron overload is often present in MDS patients. We hypothesize that decitabine may play a therapeutic role by inducing ferroptosis in MDS cells through the increase of iron-dependent ROS. This study was to investigate whether ferroptosis involves in the pathogenesis of MDS and whether decitabine could induce ferroptosis in MDS cells.

## Materials and Methods

### Reagents

Fer-1 and dimethyl sulfoxide (DMSO) were purchased from Sigma Chemical (United States). The 6-carboxy-2’,7’dichlorodihydrofluorescein diacetate (DCFH-DA) and Z-VAD-FMK were purchased from Beyotime Institute of Biotechnology (China); FeRhoNox-1 was purchased from GORYO Chemical (Japan); erastin was purchased from Selleck Chemical (United States); Roswell Park Memorial Institute 1640 (RPMI 1640) was purchased from Gibco (United States); deferoxamine mesylate was purchased from TargetMol (United States); fetal bovine serum (FBS) was purchased from Lonsera (Uruguay); Hank’s balanced salt solution (HBSS) was purchased from Macgene (China); phosphate-buffered saline (PBS); and lymphocyte separation medium were purchased from Solarbio (China); iron dextran was purchased from Pharmacosmos A/S (Denmark); and necrostatin-1 was purchased from Santa Cruz (United States).

### Cell Culture Experiments

Two cell lines (SKM-1 and MUTZ-1) were cultured in RPMI 1640 medium containing 10% FBS, 100-μg/ml penicillin (Gibco), and 100-U/ml streptomycin (Gibco) in a 5% CO_2_/95% air incubator at 37°C. SKM-1 was obtained from the Health Science Research Resources Bank in Japan (25). MUTZ-1, a cell line established from childhood MDS with excess blasts, was obtained from the German Braunschweig Cell Center (26).

### Cell Viability Assay (CCK-8 Assay)

Cell viability was measured by CCK-8 assay (Beyotime, China). Cells were seeded into 96-well plates at 3 × 10^5^ cells/ml in 200-μl complete medium and cultured in the complete medium at 37°C for 24 h in a 5% CO_2_/95% air incubator. Then, the cells were treated with decitabine with or without inhibitors in the complete medium for the indicated times. Then, 20 μl of CCK-8 reagent was added to the wells and incubated for 4 h. The absorption at 450 nm was measured to calculate cell viability. The experiment was repeated three times, and each sample had three duplications. Cell viability was calculated using the following equation: proliferation (%) = (OD450 of isogarcinol group/OD450 of the control group) × 100%.

### Detection of Intracellular Reactive Oxygen Species

Intracellular ROS was detected using an oxidation-sensitive fluorescent probe (DCFH-DA). After treatment with 100-nM decitabine, with or without Fer-1 (0.4 μM), desferrioxamine (DFO) (50 μM), or erastin (10 μM) for 48 h, cells were washed twice in PBS. They were then incubated with 5-μmol/L DCFH-DA at 37°C for 20 min according to the manufacturer’s instructions. DCFH-DA was deacetylated intracellularly by nonspecific esterase, which was further oxidized by ROS to the fluorescent compound 2,7-dichlorofluorescein (DCF). DCF fluorescence was detected by FACScan flow cytometer (Becton Dickinson). For each sample, 10,000 events were collected.

### Intracellular Glutathione and Glutathione Peroxidase 4 Activity Assay

The GSH level was measured by reacting on 5,5’-dithiobis(2-nitrobenzoic acid) to form a yellow substance, which can be determined by colorimetry. It was determined with the GSH and GSSG Assay Kit (Beyotime, China) (27). The GPX4 activity [nanomole nicotinamide adenine dinucleotide phosphate (NADPH) per minute per milliliter] was measured in the supernatant using a cellular GPX assay kit (Beyotime Institute of Biotechnology) that measures the coupled oxidation of NADPH during GSH reductase recycling of oxidized GSH from GPX-mediated reduction of t-butyl peroxide. In the assay, excess GSH reductase, GSH, and NADPH were added according to the manufacturer’s instruction.

### Mice and Treatment

C57BL/6 mice were purchased from Vital River (Beijing, China) at 6 to 8 weeks of age. The animals were quarantined and allowed to acclimatize for 1 week. They were maintained in a room at 22 ± 2°C, with a relative humidity of 50 ± 10%. Twenty mice were housed with five individuals per cage and used at a weight of approximately 20.0–22.0 *g*. They were randomly divided into four groups, five in each group, namely control group, low-dose group, middle-dose group, and high-dose group. The low-, middle-, and high-dose group mice were administered an intraperitoneal injection of 0.2-ml iron dextran at a concentration of 6.25, 12.5, and 25 mg/ml, respectively, every 3 days for 10 weeks to establish iron overload model. At the same time, normal saline was given to the control group.

### Isolation of Bone Marrow Mononuclear Cells in Mice

Bone marrow mononuclear cells (BMMNCs) were flushed from the femurs of mice into homogenate rinse fluid (Tbdscience, China). The cell suspension was loaded on lymphocyte separation medium (Tbdscience, China) and centrifuged for 25 min at 450 *g*. BMMNCs were isolated from the layer between the lymphocyte separation medium and blood plasma and washed three times in PBS (Solarbio, China). The isolated BMMNCs were then cultured in RPMI 1640 medium containing 10% FBS.

### Isolation of Bone Marrow Mononuclear Cells in Patients

A total of 5 ml of bone marrow aspirate was obtained from each patient. BMMNCs were isolated by density gradient centrifugation. The cell suspension was loaded on lymphocyte separation medium and centrifuged for 20 min at 2,200 rpm/min. BMMNCs were isolated from the layer between the lymphocyte separation medium and blood plasma and washed three times in PBS. The isolated BMMNCs were then cultured in RPMI 1640 medium containing 10% FBS.

### Detection of Ferritin

The ferritin was detected using enzyme-linked immunosorbent assays (ELISAs). Mouse ferritin ELISA kit was purchased from Abnova (Taiwan). ELISAs were performed according to the manufacturer’s protocol.

### Detection of Ferrous Iron

We used FeRhoNox-1 to detect intracellular ferrous iron. The isolated BMMNCs were washed with HBSS three times and incubated with FeRhoNox-1 in HBSS (5 μM) in a dark, humidified container at room temperature for 60 min. After washing with HBSS three times, the section was covered with a small drop of the mounting medium and observed using fluorescence microscopy (Leica, Germany) or quantitatively measured using a FACScan flow cytometer (Becton Dickinson, United States).

### Statistical Analysis

Result analysis was performed with the GraphPad Prism 8.0 program (GraphPad Software, Inc., San Diego, CA, United States). Data that obeyed normal distribution were presented as means ± SD, and multiple group comparisons were performed using a one-way analysis of variance. The analysis of correlation was performed by linear regression. A *p*-value of <0.05 was considered statistically significant.

## Results

### Decitabine Triggers Ferroptosis and Necroptosis in Myelodysplastic Syndrome Cells

**FIGURE 1:**
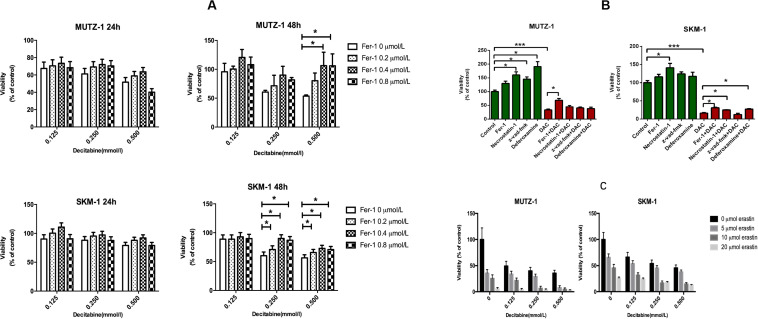
Decitabine induces ferroptosis in myelodysplastic syndrome cells and its mechanism. **(A)** Ferroptosis induced by decitabine under different concentrations and duration. CCK-8 assay of cell viability in MUTZ-1 and SKM-1 cell lines. Data are mean ± SE; *n* = 5. *P < 0.05 by ANOVA/Bonferroni. ANOVA, analysis of variance; SE, standard error. **(B)** Effects of different inhibitors on cytotoxicity of decitabine. CCK-8 assay of cell viability in MUTZ-1 and SKM-1 cell lines. DAC, 0.5 mM; Fer-1, 0.4 μM; necrostatin-1, 30 μM; Z-VAD-FMK, 20 μM; and deferoxamine, 50 μM. Data are mean ± SE; *n* = 5. *P < 0.05, by ANOVA/Bonferroni. ANOVA, analysis of variance; DAC, decitabine; and SE, standard error. **(C)** Erastin enhances the inhibitory effect of decitabine on MDS cell lines. CCK-8 assay of cell viability in MUTZ-1 and SKM-1 cell lines. Data are mean ± SE; *n* = 5. ***P < 0.001.

To determine whether ferroptosis is triggered by decitabine in MDS cells, two cell lines (SKM-1 and MUTZ-1) were treated with decitabine (0.125, 0.250, and 0.500 mM) and Fer-1 (0.2, 0.4, and 0.8 μM) for 24 or 48 h, respectively. CCK-8 determined cell proliferation. The result showed that Fer-1 protected MDS cells from being inhibited by decitabine. The viability of SKM-1 and MUTZ-1 cells was significantly inhibited by decitabine after 48-h treatment at the concentration of 0.5 mM. Fer-1 had the most obvious protective effect at 0.4 μM (Figure 1A). To determine if ferroptosis, necroptosis, and apoptosis are primed in MDS cells, two cell lines were treated with decitabine (0.5 mM) alone or in combination with Fer-1 (0.4 μM), necrostatin-1 (30 μM), Z-VAD-FMK (20 μM), or DFO (50 μM) for 48 h. The result showed that necrostatin-1 and Z-VAD-FMK increased the cell viability of MUTZ-1 significantly. With the effect of Fer-1 being the most significant (Figure 1B), necrostatin-1 increased the cell viability of SKM-1 significantly. Fer-1, DFO, and necrostatin-1 could partially reverse the growth-inhibitory effect of decitabine on SKM-1 and MUTZ-1, showing decitabine induced ferroptosis and necroptosis in MDS cells. MUTZ-1 and SKM-1 were next treated with decitabine (0, 0.125, 0.250, and 0.500 mM) in combination with erastin (0, 5, 10, and 20 μM) for 48 h (Figure 1C). The results showed that erastin could increase the cytotoxicity of decitabine at different concentrations in MUTZ-1 and SKM-1 cells. The synergistic value was not significant, as shown in Supplementary Table 1 (*Q* < 1.15), indicating that erastin displayed an additive effect with decitabine in suppressing viability of MDS cells.

### Treatment With Decitabine Increases Intracellular Reactive Oxygen Species Level by Reducing Glutathione Level and Glutathione Peroxidase 4 Activity in Myelodysplastic Syndrome Cells

**FIGURE 2:**
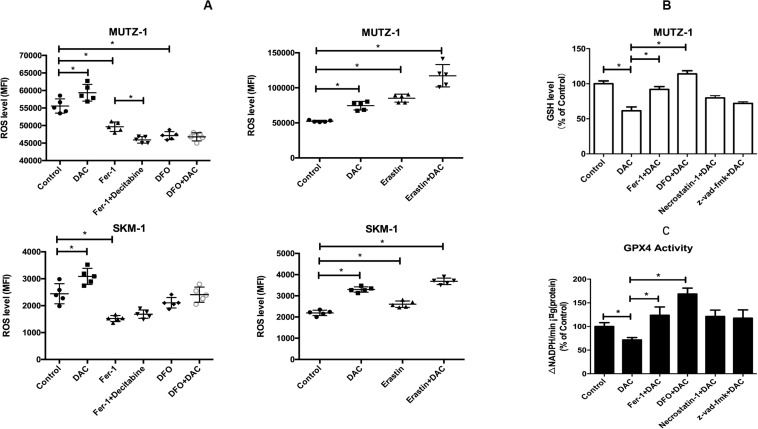
Mechanism of decitabine in inducing ferroptosis. **(A)** Effect of decitabine on intracellular ROS level in MUTZ-1 and SKM-1 cell lines by flow cytometry. Decitabine, 100 nM; Fer-1, 0.4 μM; necrostatin-1, 30 μM; Z-VAD-FMK, 20 μM; DFO, 50 μM; and erastin, 10 μM. Mean, mean fluorescence intensity (MFI). **(B)** Effect of decitabine on GSH level in MDS cells. GSH and GSSG assay of GSH level in the MUTZ-1 cell line. Decitabine, 0.5 mM; Fer-1, 0.4 μM; and DFO, 50 μM. Data are mean ± SE; *n* = 3. *P < 0.05 by ANOVA/Bonferroni. ANOVA, analysis of variance; DAC, decitabine; and SE, standard error. **(C)** Effect of decitabine on GPXs activity in MDS cells. Cellular glutathione peroxidase assay of GPXs activity in the MUTZ-1 cell line. DAC, 0.5 mM; Fer-1, 0.4 μM; GSH, 1 mM; necrostatin-1, 30 μM; and Z-VAD-FMK, 20 μM. Data are mean ± SE; *n* = 3. *P < 0.05 by ANOVA/Bonferroni. ANOVA, analysis of variance; SE, standard error.

The production of ROS played an important role in ferroptosis; the level of ROS was measured by flow cytometry to test whether decitabine induced ferroptosis. Figure 2A shows that decitabine (100 nM) led to a remarkable increase in intracellular ROS, which could be inhibited by Fer-1 and DFO whereas promoted by erastin.

We next checked changes in the GSH level upon decitabine treatment. MUTZ-1 cell line was treated with decitabine (0.5 mM) alone or in combination with Fer-1 (0.4 μM) or DFO (50 μM) for 48 h. Figure 2B shows that decitabine led to a remarkable depletion of cellular GSH compared with the control, which could be inhibited by Fer-1 and DFO significantly.

Glutathione peroxidase 4 activity was further measured. MUTZ-1 cell line was treated with decitabine (0.5 mM) alone or in combination with Fer-1 (0.4 μM), GSH (1 mM), necrostatin-1 (30 μM), or Z-VAD-FMK (20 μM) for 48 h. Figure 2C shows that decitabine led to a significant reduction of GPX4 activity, which could be inhibited by Fer-1, GSH, necrostatin-1, and Z-VAD-FMK significantly.

### Establishment of a Mouse Model of Iron Overload

**FIGURE 3:**
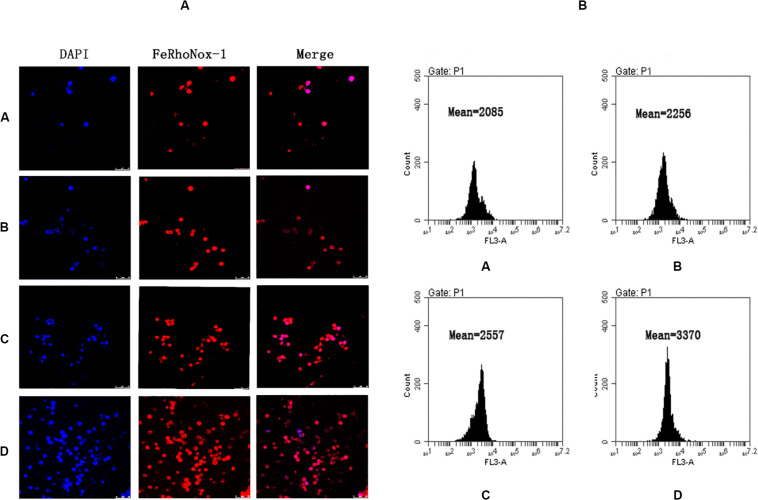
Establishment of a mouse model of iron overload. **(A)** Intracellular ferrous ions in BMMNCs by fluorescence microscopy. FeRhoNox-1 was used to detect the intracellular ferrous ions by fluorescence microscopy in BMMNCs. FeRhoNox-1, an activatable fluorescent probe that specifically detects labile Fe^2+^ ions via orange (red) fluorescence, 5 μM. DAPI, 4,6-diamidino-2-phenylindole, a blue fluorescent nucleic acid stain that preferentially stains double-stranded DNA (dsDNA). BMMNCs, bone marrow mononuclear cells. A, control group; B, low-dose iron group; C, middle-dose iron group; and D, high-dose iron group. **(B)** Amount of intracellular ferrous ions in BMMNCs by flow cytometry. Flow cytometry was used to detect the intracellular ferrous ions in BMMNCs of four groups, quantitatively. A, control group; B, low-dose iron group; C, middle-dose iron group; and D, high-dose iron group. Mean, mean fluorescence intensity (MFI).

We examined both the external form and pathological changes in the liver, spleen, and femur. The livers of the mice in the iron overload group showed marked enlargement, congestion, and edema (Supplementary Figure 1A), so were the spleens (Supplementary Figure 1B). Large amounts of iron deposition could be observed in the bone marrow, spleen, and liver of iron overload groups compared with the control group (Supplementary Figure 1C). This showed that the administration of iron dextran by intraperitoneal injection led to large amounts of iron deposition in organs and tissues, resulting in spleen and liver enlargement, thus establishing an iron overload model in C57BL/6 mice. Also, confocal microscopy was used to observe the amount of intracellular ferrous ions in BMMNCs of the four groups (Figure 3A). The average fluorescence intensity of each group was quantitatively measured by flow cytometry. The results showed that the level of Fe2^+^ had an increasing trend in the four groups (Figure 3B), so was the level of ferritin (Supplementary Figure 1D). The differences of ferritin between the iron overload groups and the control group were also significant, indicating that iron overload was established successfully (Supplementary Figure 1D).

### Iron Overload Induces Ferroptosis in Mice

**FIGURE 4:**
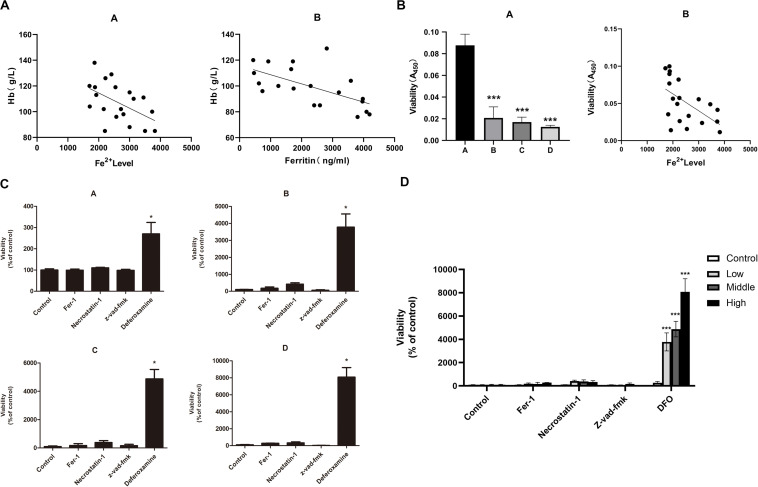
**(A)** Correlation between the degree of anemia and iron overload in mice. A, correlation between hemoglobin concentration and intracellular Fe^2+^ level. *r*^2^ = 0.2996, and *P* < 0.05 by linear regression. B, correlation between hemoglobin concentration and ferritin concentration in mice. *r*^2^ = 0.3274, *P* < 0.05 by linear regression. **(B)** Correlation between the cell viability of BMMNCs and iron overload. A, CCK-8 assay of cell viability in four groups. A, control group; B, low-dose iron group; C, middle-dose iron group; and D, high-dose iron group. Data are mean ± SE; *n* = 3. *P < 0.05 by ANOVA/Bonferroni compared with the control group. ANOVA, analysis of variance; SE, standard error. B, correlation between the cell viability of BMMNCs and the level of Fe^2+^ in mice. *r*^2^ = 0.3117, *P* < 0.05 by linear regression. **(C)** Effects of different inhibitors on BMMNCs of mice. CCK-8 assay of cell viability of four groups. A, control group; B, low-dose iron group; C, middle-dose iron group; and D, high-dose iron group. Fer-1, 0.4 μM; necrostatin-1, 30 μM; Z-VAD-FMK, 20 μM; and deferoxamine, 50 μM. Data are mean ± SE; *n* = 4. *P < 0.05, by ANOVA/Bonferroni compared with the control. ANOVA, analysis of variance; SE, standard error. **(D)** The comparison of cell viability among four groups of BMMNCs. ***P < 0.001 by ANOVA/Bonferroni compared with the control group. ANOVA, analysis of variance.

**FIGURE 5:**
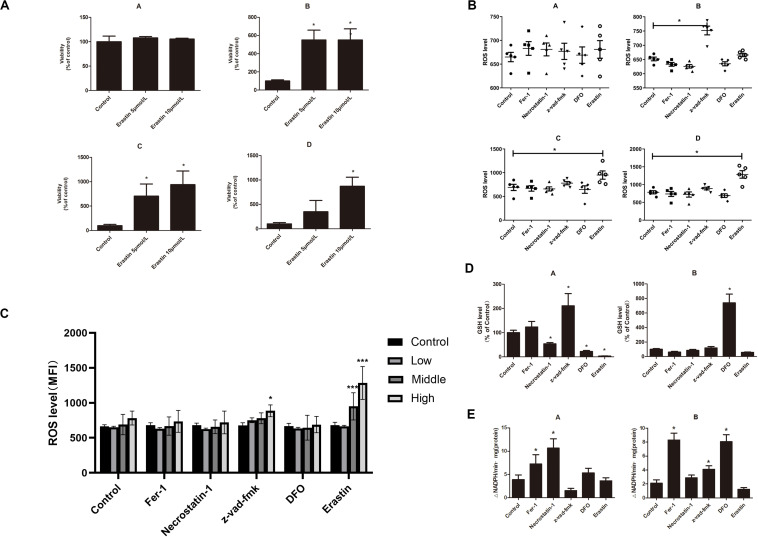
**(A)** Effects of erastin on BMMNCs of mice. CCK-8 assay of cell viability of four groups. A, control group; B, low-dose iron group; C, middle-dose iron group; and D, high-dose iron group. Data are mean ± SE; *n* = 3. *P < 0.05 by ANOVA/Bonferroni compared with the control. ANOVA, analysis of variance; SE, standard error. **(B)** Intracellular ROS level in BMMNCs of mice. Level of ROS in BMMNCs of mice by flow cytometry. A, control group; B, low-dose iron group; C, middle-dose iron group; and D, high-dose iron group. Fer-1, 0.4 μM; necrostatin-1, 30 μM; Z-VAD-FMK, 20 μM; DFO, 50 μM; and erastin, 10 μM. Data are mean fluorescence intensity (MFI) ± SE; *n* = 3. *P < 0.05 by ANOVA/Bonferroni compared with the control. ANOVA, analysis of variance; SE, standard error. **(C)** Comparison of intracellular ROS level and GPXs activity in the control group and high-dose iron group. *P < 0.05 by unpaired *t*-test. MFI, mean fluorescence intensity. **(D)** GSH level in BMMNCs of mice. GSH levels of the control group and high-dose iron group were detected by GSH and GSSG assay. A, control group; B, high-dose iron group. Fer-1, 0.4 μM; necrostatin-1, 30 μM; Z-VAD-FMK, 20 μM; DFO, 50 μM; and erastin, 10 μM. Data are mean fluorescence intensity (MFI) ± SE; *n* = 3. *P < 0.05 by ANOVA/Bonferroni compared with the control. ANOVA, analysis of variance; SE, standard error. **(E)** Activity of GPXs in BMMNCs of mice. Activity of GPXs of the control group and high-dose iron group were detected by cellular glutathione peroxidase assay. A, control group; B, high-dose iron group. Fer-1, 0.4 μM; necrostatin-1, 30 μM; Z-VAD-FMK, 20 μM; DFO, 50 μM; and erastin, 10 μM. Data are mean fluorescence intensity (MFI) ± SE; *n* = 3. *P < 0.05 by ANOVA/Bonferroni compared with the control. ANOVA, analysis of variance; SE, standard error. ***P < 0.001.

The iron overload model of C57BL/6 mice was constructed to observe if iron overload could induce ferroptosis. The results demonstrated that the hemoglobin of the high-dose iron group was significantly lower than that of the control group (Supplementary Table 2), indicating that iron overload could lead to anemia in mice. Also, the concentration of hemoglobin was negatively correlated with ferritin concentration and intracellular Fe^2+^ level (Figure 4A). Moreover, iron overload inhibited the proliferation of BMMNCs, which was negatively correlated with the intracellular Fe^2+^ level (Figure 4B). Fer-1 and necrostatin-1 partially mitigated the decrease of cell proliferation in the iron overload groups (Figure 4C), whereas erastin promoted the proliferative activity of BMMNCs in the iron overload mice (Figure 5A). Compared with the control, the activity of GPX4 and the level of GSH decreased, whereas the level of ROS increased in BMMNCs of the iron overload mice (Figures 5B–E). DFO increased the level of GSH in the iron overload mice (Figure 5D). Fer-1, DFO, and Z-VAD-FMK increased the GPX4 activity of BMMNCs in the iron overload mice (Figure 5E).

### Differential Mechanisms Induced by Decitabine Between Low- and High-Risk Myelodysplastic Syndrome Patients

**FIGURE 6:**
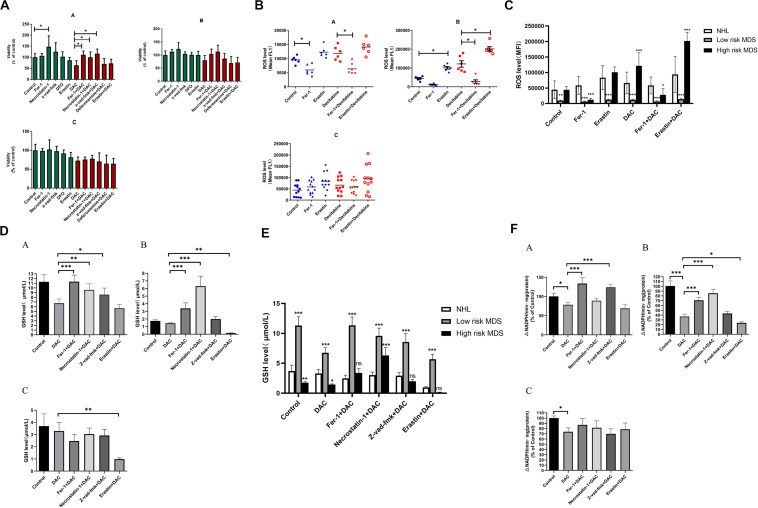
Decitabine induces ferroptosis in bone marrow mononuclear cells of patients with MDS. **(A)** Cell viability of patients was detected by CCK-8 assay. A, low-risk MDS patients; B, high-risk MDS patients; and C, lymphoma patients. DAC, 0.5 mM; Fer-1, 0.4 μM; necrostatin-1, 30 μM; Z-VAD-FMK, 20 μM; deferoxamine, 50 μM; and erastin, 10 μM. Data are mean ± SE; *n* = 3. *P < 0.05, by ANOVA/Bonferroni. ANOVA, analysis of variance; DAC, decitabine; and SE, standard error. **(B)** Level of ROS in BMMNCs of MDS patients. Level of ROS in BMMNCs of patients by flow cytometry. A, low-risk MDS patients; B, high-risk MDS patients; and C, lymphoma patients. Decitabine, 100 nM; Fer-1, 0.4 μM; and erastin, 10 μM. Data are mean ± SE; *n* = 3. *P < 0.05 by ANOVA/Bonferroni. ANOVA, analysis of variance; SE, standard error. **(C)** Comparison of intracellular ROS levels among the three groups of patients. *P < 0.05 by two-way ANOVA/Bonferroni compared with the control group. ANOVA, analysis of variance; MFI, mean fluorescence intensity. **(D)** Level of GSH in BMMNCs of MDS patients. GSH levels of patients were detected by GSH and GSSG assay. A, low-risk MDS patients; B, high-risk MDS patients; and C, lymphoma patients. DAC, 0.5 mM; Fer-1, 0.4 μM; necrostatin-1, 30 μM; Z-VAD-FMK, 20 μM; and erastin, 10 μM. Data are mean ± SE; *n* = 3. *P < 0.05 by ANOVA/Bonferroni. ANOVA, analysis of variance; DAC, decitabine, and SE, standard error. **(E)** Comparison of GSH levels among the three groups of patients. *P < 0.05 by two-way ANOVA/Bonferroni compared with the control group. ANOVA, analysis of variance; ns, no significance. **(F)** Activity of GPXs in BMMNCs of MDS patients. Cellular glutathione peroxidase assay detected the activity of GPXs of patients. A, low-risk MDS patients; B, high-risk MDS patients; and C, lymphoma patients. DAC, 0.5 mM; Fer-1, 0.4 μM; necrostatin-1, 30 μM; Z-VAD-FMK, 20 μM; and erastin, 10 μM. Data are mean ± SE; *n* = 3. *P < 0.05 by ANOVA/Bonferroni. ANOVA, analysis of variance; DAC, decitabine; and SE, standard error. **P < 0.01, ***P < 0.001.

To study the role of ferroptosis in the pathogenesis of MDS patients, the BMMNCs were obtained from 6 low-risk MDS patients, 6 high-risk MDS patients, and 12 controls (non-Hodgkin lymphoma without bone marrow involvement) and co-cultured with the earlier mentioned inhibitors and decitabine. The results indicated that Fer-1, Z-VAD-FMK, and necrostatin-1 significantly reversed the inhibitory effect of decitabine on cell viability in low-risk MDS patients (Figure 6A). Necrostatin-1 and Fer-1 significantly reversed the inhibitory effect of decitabine in high-risk MDS patients (Figure 6A). Decitabine significantly increased the ROS level in both MDS groups, which could be inhibited by Fer-1 or promoted by erastin (Figures 6B,C). Fer-1, necrostatin-1, and Z-VAD-FMK could significantly reverse the inhibitory effect of decitabine on GSH levels in low-risk MDS patients (Figures 6D,E). Fer-1 and necrostatin-1 could significantly reverse the inhibitory effect of decitabine on GSH levels in high-risk MDS patients (Figures 6D,E). Erastin combined with decitabine could further reduce the GSH level, and the difference was significant in the high-risk MDS group (Figures 6D,E). For low-risk MDS, GPX4 activity of the groups treated with Fer-1 plus decitabine and Z-VAD-FMK plus decitabine was significantly higher than that of the group treated with decitabine only (Figure 6F). For high-risk MDS, GPX4 activity of the groups treated with Fer-1 plus decitabine and necrostatin-1 plus decitabine was significantly higher than that of the sole decitabine group (Figure 6F). This demonstrated that the suppressive effect of decitabine on GPX4 activity was significantly reversed by Fer-1 and Z-VAD-FMK in low-risk MDS whereas antagonized by Fer-1 and necrostatin-1 in high-risk MDS (Figure 6F). Erastin could further decrease the activity of GPX4 when compared with the decitabine group; the difference was significant in the high-risk MDS group (Figure 6F). Decitabine had a little effect on BMMNCs from the non-Hodgkin lymphoma, but it is not as obvious as MDS.

## Discussion

Ferroptosis is a regulated form of cell death involving iron-dependent loss of GPX4 activity and subsequent accumulation of lipid peroxides (4, 7). Cells undergoing ferroptosis cannot be rescued by chemical or genetic inhibitors of apoptosis (such as Z-VAD-FMK) (28–31) or inhibitors of necroptosis (such as Nec-1 or RIPK1/3 knockdown) (4, 32), which suggests that ferroptosis is a distinct cell death form. Inhibition of ferroptosis is shown to be protective in models of Huntington’s disease (33), periventricular leukomalacia (33–35), and kidney dysfunction (33, 36, 37). Mice that are depleted of GPX4 do not survive over embryonic day 8, showing that protection from ferroptosis is essential during normal mammalian development. There are also studies showing that induction of ferroptosis could inhibit tumor growth in a xenograft mouse model of fibrosarcoma, indicating that inducing ferroptosis may be a potential anticancer treatment. It has been shown that GPX4 and system Xc^–^ are required for the survival of certain tumor cells, including renal cell carcinoma and diffuse large B cell lymphoma cells, and a characteristic ferroptotic death is observed in these cells upon GPX4 knockdown.

Myelodysplastic syndrome is the most common acquired adult bone marrow failure syndrome, which is often accompanied by iron overload. Because ferroptosis is an iron-dependent process, we established an iron overload model using C57BL/6 mice to observe whether iron overload could induce ferroptosis, aiming at providing new ideas for the treatment of iron overload MDS patients. Zhang et al. (38) established an iron overload mouse model by injection with iron dextran intraperitoneally and found that iron overload could impair the bone marrow microenvironments to inhibit hematopoiesis. Jin et al. (39) used the RUNX mutant vector with iron dextran to construct an iron overload mouse model and found hematopoiesis was inhibited by iron overload with ROS increased. MDS is a group of highly heterogeneous diseases, most of which are associated with multiple gene mutations, such as TET2, DNMT3A, ASXL1, SF3B1, and SRSF1. Multiple MDS mouse models have been constructed using genetic engineering methods, which provided advantageous platforms for in-depth study of MDS. However, these models are difficult to recapitulate most of the biological characteristics of human MDS (40–46). Our mouse model mimics iron overload well, but they lack bone marrow failure of MDS. This is a limitation of this study. Fortunately, recently, MDS patient-derived xenograft technology develops quickly, which provides a good tool for better simulation of human MDS in the future (47).

The Food and Drug Administration of the United States approved decitabine for the treatment of high-risk MDS or leukemia unfit for intensive chemotherapy (48). A low dose of decitabine can induce differentiation, suppress proliferation, and trigger apoptosis through inducing re-expression of certain functional genes. High-dose decitabine is known to display cytotoxic effects. Recently, researchers have found that there was no correlation between methylation reversal of tumor suppressor genes and clinical response in some patients treated with decitabine, implying that demethylation drugs might have other unidentified mechanisms that need to be elucidated (49, 50). The results of our study showed that Fer-1, DFO, necrostatin-1, and the former that has the most significant effect could reverse the growth-inhibitory effect of decitabine on SKM-1 and MUTZ-1, indicating that ferroptosis and necroptosis may be the main mechanisms of decitabine-induced cell death of MDS cells. Besides, our results also showed that decitabine-induced ROS raise led to ferroptosis in MDS cells by decreasing GSH level and GPX4 activity, which further clarified the mechanism of ferroptosis induced by decitabine.

Next, with the iron overload mouse model, we demonstrated that iron overload was able to induce ferroptosis, which subsequently caused anemia. Besides, we found that erastin significantly increased the viability of BMMNCs in iron overloaded mice unexpectedly, which puzzled us. Wang et al. (51) also showed that the ferroptosis inducer erastin did not cause cell death in human peripheral blood mononuclear cells. On the contrary, erastin-induced lipid peroxidation promoted human peripheral blood mononuclear cell proliferation and differentiation into B cells and natural killer cells by suppressing bone morphogenetic protein family. These findings uncover a potential new function of erastin in promoting mononuclear cells’ proliferation and differentiation, which needs further studies. Our results further showed that the increase of ROS level led by iron overload was related to ferroptosis and necroptosis, which might account for the main forms of cell death in iron overload mice. The iron overload mouse model partially mimicked the anemia observed in MDS patients, especially those with iron overload, and further demonstrated the mechanism of ferroptosis in this context.

To further investigate whether decitabine has differential mechanisms in low- and high-risk MDS patients, we conducted additional experiments using primary cells from MDS patients. The results showed that inhibitors of ferroptosis and apoptosis compromised the suppressive effect of decitabine on GPX4 in low-risk MDS, whereas inhibitors of ferroptosis and necrosis antagonized decitabine’s suppression on GPX4 in high-risk MDS. These data indicated that decitabine mainly induced ferroptosis and apoptosis in low-risk MDS patients and necroptosis and ferroptosis in high-risk MDS patients. In low-risk MDS, the tumor burden is relatively low. BMMNC is mainly dominated by non-tumor cells, and peripheral blood is mainly manifested by cytopenia. Decitabine induces ferroptosis and apoptosis, which will exacerbate the severity of cytopenia. Therefore, the use of standard doses of decitabine for low-risk MDS should be very cautious and needs further exploration. In high-risk MDS, decitabine can induce ferroptosis and necroptosis, kill MDS cloned cells, restore normal hematopoiesis, and delay the transformation to AML.

There are several limitations to our paper. First, SKM-1 and MUTZ-1 cell lines cannot completely represent the traits of MDS primary cells, and the *in vitro* culture conditions cannot also represent changes in the bone marrow microenvironment. Second, the iron overload mouse lacks a background of MDS bone marrow failure and cannot fully mimic the pathological state of iron overload in MDS patients.

Our findings give a better understanding of ferroptosis induced by decitabine and its molecular mechanism in MDS cells, gaining insight into ferroptosis-mediated cancer treatment.

## Data Availability Statement

All datasets presented in this study are included in the article/Supplementary Material.

## Ethics Statement

The studies involving human participants were reviewed and approved by the Ethics Committee of Tianjin Medical University General Hospital. The patients/participants provided their written informed consent to participate in this study. The animal study was reviewed and approved by The Ethics Committee of Tianjin Medical University General Hospital.

## Author Contributions

QL and HN performed research and analyzed the data. HW designed the research, ensured correct analysis of the data, and wrote of the manuscript. LaY, JL, LiY, HJ, CL, ZS, and LX assisted in the design of the research, oversaw the collection of the data, and contributed to the writing of the manuscript. All authors critically revised the manuscript and gave final approval of the manuscript.

## Conflict of Interest

The authors declare that the research was conducted in the absence of any commercial or financial relationships that could be construed as a potential conflict of interest.
