# Supplementary material for: Abnormal Ferroptosis in Myelodysplastic Syndrome
Source: Front Oncol. 2020 Sep 2;10:1656. doi: 10.3389/fonc.2020.01656 (PMC7492296; doi:10.3389/fonc.2020.01656)
Supplement: Supplementary file 1 [file Data_Sheet_1.docx]

**Supplemental figures**

**figure 1.** **Establishment of a mouse model of iron overload. a, b, c. Iron deposition observed in the liver, spleen and BM of C57BL/6 mice**. (A) The livers of mice. A, the control group, B, the low dose iron group, C, the middle dose iron group, D, the high dose iron group. (B) The spleens of mice. A, the control group, B, the low dose iron group, C, the middle dose iron group, D, the high dose iron group. (C) The pathological changes of the liver, spleen and femur. Arrow(white), brown iron deposition by HE staining. Arrow(black), blue iron deposition by Prussian blue staining. BM, bone marrow. (D)**.** The level of ferritin. The level of ferritin in four groups of mice was detected by ELISA. Data are mean±SE; n≥3. **P*<0.05 by ANOVA/Bonferroni. ANOVA, analysis of variance, SE, standard error.

1. **
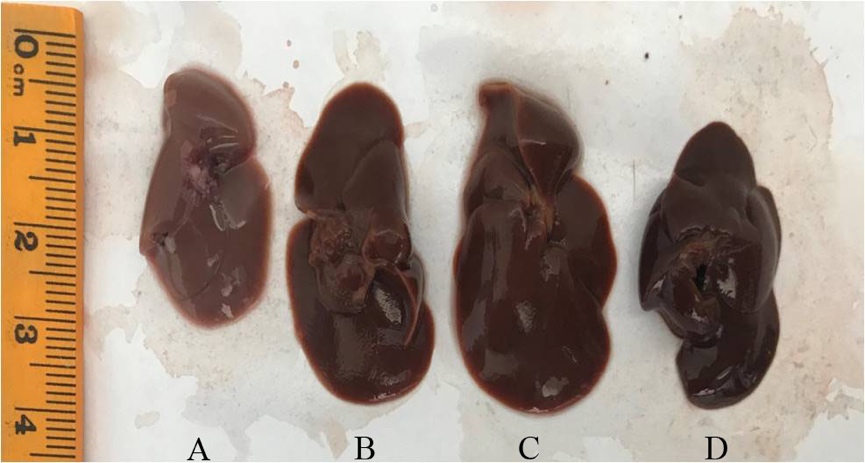
**
2. **
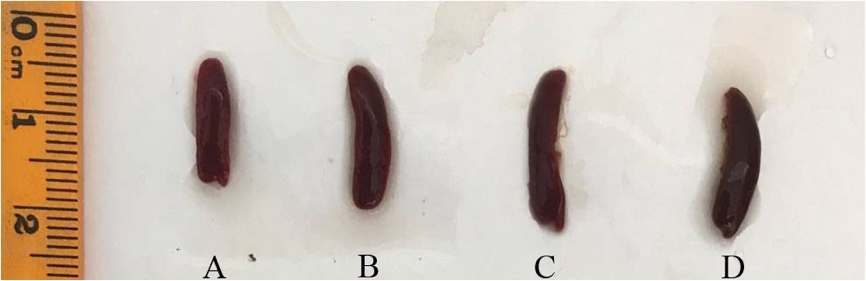
**
3. **
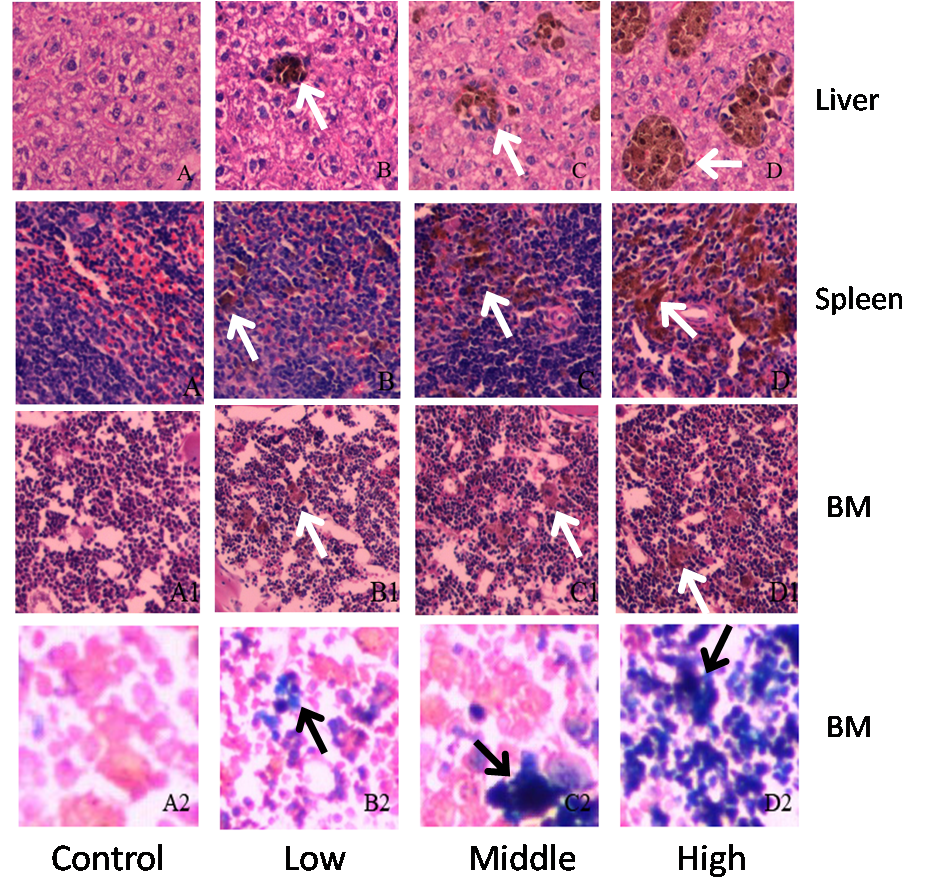
**
4. **
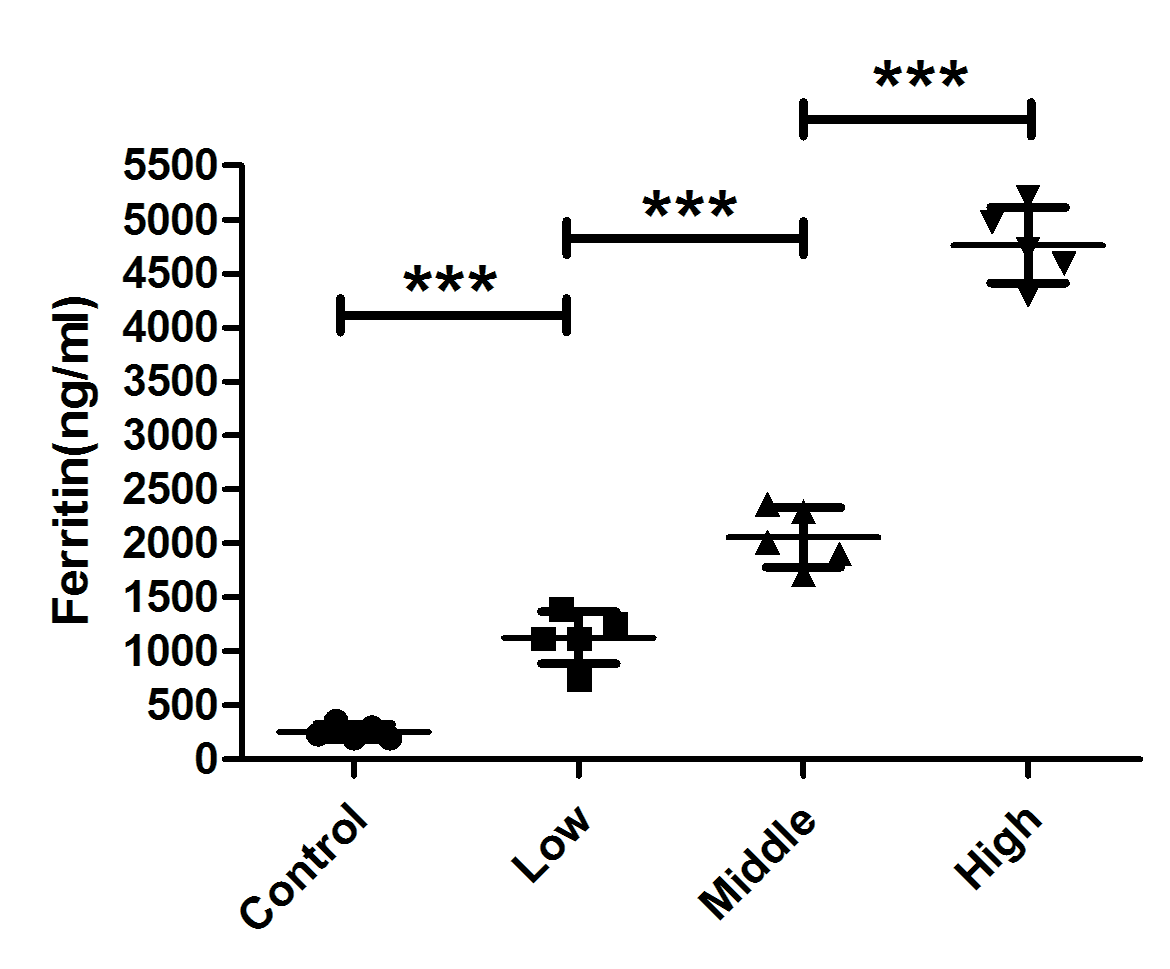
**
