# Supplementary material for: Abnormal Ferroptosis in Myelodysplastic Syndrome
Source: Front Oncol. 2020 Sep 2;10:1656. doi: 10.3389/fonc.2020.01656 (PMC7492296; doi:10.3389/fonc.2020.01656)
Supplement: Supplementary file 2 [file Data_Sheet_2.docx]

**Supplemental Tables**

**Table 1.** **Inhibitory effect of Erastin combined with decitabine on MUTZ-1 cells**. CCK-8 assay of cell viability in MUTZ-1 cell line. Cell viability = A _experimental group_/A _control group_ ×100%; Inhibition rate (IR) = (1-A _experimental group_/A _control group_) ×100%. Drug combination fraction (Q value)=IR_A+B_/(IR_A_+IR_B_-IR_A_*IR_B_), Q>1.15 is considered to have significant synergistic effect. IR_A_, the inhibitory rate of drug A, IR_B_, the inhibitory rate of drug B, IR_A+B_, combined inhibitory effect of drug A and drug B.

**Table 2. The count of peripheral blood cells of mice in each group.** The count of peripheral blood cells is detected by automatic hematology analyzer (NIHON KOHDEN CORP.). (A) The control group. (B) The low dose iron group. (C) The middle dose iron group. (D) The high dose iron group. Data are mean±SD; n=3. **P*< .05 by ANOVA/Bonferroni compared with the control. ANOVA, analysis of variance, SD, Standard Deviation.

**Table 1**

**
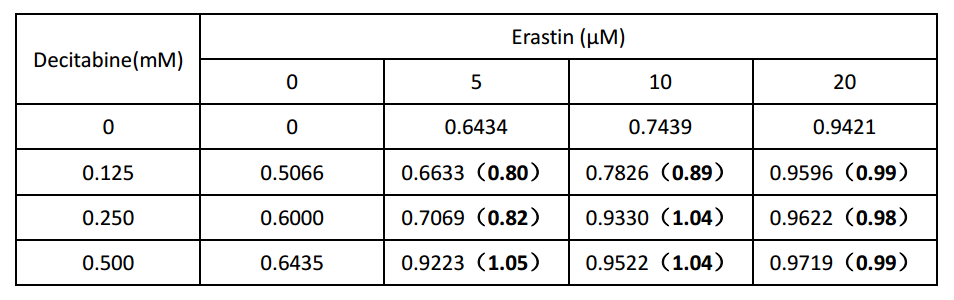
**

**Table 2**

**
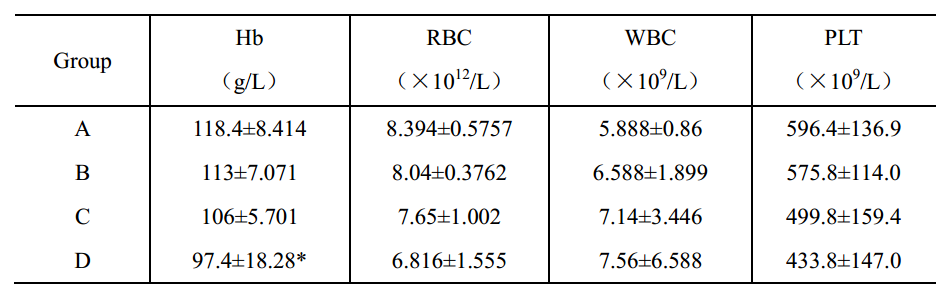
**
